# Toll-like receptor signaling pathway triggered by inhibition of serpin A1 stimulates production of inflammatory cytokines by endometrial stromal cells

**DOI:** 10.3389/fendo.2022.966455

**Published:** 2022-08-24

**Authors:** Kazuya Kusama, Ayaka Satoyoshi, Mana Azumi, Mikihiro Yoshie, Junya Kojima, Yumi Mizuno, Masanori Ono, Hirotaka Nishi, Takeshi Kajihara, Kazuhiro Tamura

**Affiliations:** ^1^ Department of Endocrine Pharmacology, Tokyo University of Pharmacy and Life Sciences, Tokyo, Japan; ^2^ Department of Obstetrics and Gynecology, Tokyo Medical University, Tokyo, Japan; ^3^ Department of Obstetrics and Gynecology, Saitama Medical University, Saitama, Japan

**Keywords:** endometrial stromal cells, serpin A1, alpha-1 antitrypsin, toll-like receptor, interferon beta, interleukin-1 beta, endometriosis

## Abstract

Endometriosis is characterized by the presence of inflamed and fibrotic endometrial tissue outside the uterine cavity. Previously, we found decreased SERPINA1 (alpha-1 antitrypsin) expression in endometriosis-like lesions in a mouse model of endometriosis, suggesting that it exacerbated inflammation in these lesions. However, the molecular mechanism(s) by which SERPINA1 affects expression of inflammatory factors and development of endometriotic lesions have not been fully characterized. To investigate the role of intracellular SERPINA1 in endometrial stromal cells (ESCs), we performed RNA sequence analysis using RNA extracted from ESCs in which SERPINA1 was knocked down. The analysis identified several toll-like receptor (TLR)-related factors as being upregulated. Silencing of SERPINA1 increased expression of TLR3 and TLR4 in ESCs, as well as several TLR signaling pathway components, including MYD88, IRAK1/4, interleukin (IL)-1β, and interferon (IFN)-β. TLR3 or TLR4 agonists increased expression of inflammatory factors in SERPINA1-knockdown ESCs, whereas TLR3 or TLR4 inhibitors decreased expression. In addition, treatment with recombinant IL-1β or IFN-β increased expression of MYD88 and inflammatory factors in ESCs. Immunohistochemical analysis of endometriotic tissues showed that TLR3, TLR4, and MYD88 were localized in endometriosis lesions. Taken together, the data suggest that reduced expression of SERPINA1 induces expression of inflammatory factors by ESCs, which in turn are associated with TLR3/4, IL-1β, and IFN-β signaling. Regulation of intracellular SERPINA1 levels in ESCs may be a strategy to inhibit inflammatory responses in endometriotic lesions.

## Introduction

Endometriosis is a chronic inflammatory disease characterized by the presence of inflamed and fibrotic endometrial tissue outside the uterine cavity (1–4). The etiology of endometriotic lesions may be associated with retrograde menstruation, in which menstrual debris (including endometrial stromal and epithelial cells) flows back into the pelvic cavity through the fallopian tubes (1, 2). The attachment and transformation of endometrial cells at ectopic sites involves the development of endometriosis (4). Fibrogenesis and chronic inflammation, which are important stages in the formation of endometriotic lesions, are closely associated with severe pain. Several experimental models are available to study endometriosis and to evaluate the therapeutic benefits of different compounds. For example, in our experimental internal bleeding mouse model, distinct endometriosis-like grafts were generated near the surgical site of unilateral ovariectomy after transplantation of human endometrial cells. In endometriosis-like lesions, levels of interleukin 6 (IL-6) and prostaglandin E2 (PGE2) increased, whereas that of SERPINA1 (known as alpha-1 antitrypsin) decreased (5, 6). Treatment of endometrial stromal and glandular epithelial cells with PGE2 and thrombin significantly increases secretion of IL-6, as well as by endometriotic lesions (5, 7, 8).. Notably, the IL-6 and IL-8 concentrations in the serum of patients with endometriosis are higher than those in healthy women (9, 10). Furthermore, production of PGE2 is increased in patients with endometriosis (11–14).

SERPINA1, a member of the serpin superfamily, is a serine protease inhibitor synthesized primarily by hepatocytes as an acute-phase reaction product; it is then secreted into the serum (15). On the other hand, SERPINA1 is a protein ubiquitously expressed in various cells and localized in the cytoplasm and endoplasmic reticulum. Previous studies showed that a reduction in intracellular SERPINA1 protein levels exacerbates inflammatory responses in endometriosis-like lesions in mice (6), and increases endoplasmic reticulum stress-induced cytokine production by cultured human adipocytes and trophoblasts (16, 17). In addition, increased levels of inflammatory cytokines in response to lipopolysaccharide (LPS) stimulation are seen in the lungs of patients with SERPINA1 deficiency (18). By contrast, treatment with purified SERPINA1 inhibits the production of proinflammatory cytokines in human endometrial cells *in vitro* (6), and improves the survival rate of mice with peritonitis and sepsis (19). Furthermore, SERPINA1 reduces organ damage in a serine protease activity-independent fashion (17, 18, 20–22); However, the molecular mechanism(s) by which SERPINA1 affects expression of inflammatory factors likely linked to development of endometriotic lesions has not been characterized fully. Here, to investigate the physiological role of SERPINA1 in endometrial stromal cells (ESCs), we extracted RNA from ESCs in which SERPINA1 was knocked down and then performed RNA sequence analysis.

## Materials and methods

### Cell culture

Eutopic endometrial tissue samples were collected from patients with endometrioma who were undergoing surgery at Tokyo Medical University Hospital. These patients (n=3) were less than 45 years old and had regular 28–32 day menstrual cycles. Their menstrual phase (Days 16–18; early secretory phase) was identified based on their menstrual history for at least the previous 6 months. All participants provided written informed consent. The study was conducted in accordance with the principles of the Declaration of Helsinki and was approved by the Clinical Research Ethics Committee of Tokyo Medical University Hospital (approval number: 2017086). Primary cultures of endometrial cells were prepared as previously described (8). In brief, endometrial tissues from the early secretory phase were washed, minced into small pieces, and then digested for 2 h at 37°C by type I collagenase (2.5 mg/ml; Sigma-Aldrich, Tokyo, Japan), and DNase I (25 µg/ml; Nippon Gene, Tokyo, Japan). Primary ESCs and Immortalized ESC lines (23) were resuspended in Dulbecco’s modified Eagle’s medium/F12 (DMEM/F12, 1:1; Fujifilm Wako Pure Chemical Corp.) supplemented with 10% fetal bovine serum, antibiotics, and antimycotics. The ESCs were seeded onto culture dishes at 37°C in humidified air containing 5% CO_2_, and treated for 24 h with polyinosinic-polycytidylic acid (PIC, a TLR3 agonist; 10 μg/ml; Sigma-Aldrich), a TLR3/dsRNA complex inhibitor (TLR3i; 10 μM; Sigma-Aldrich), LPS (0.2 μg/ml; List Biological Labo., Campbell, CA, USA), TAK-242 (TAK, a TLR4 inhibitor; 10 μM; Selleck Chemicals, Tokyo, Japan), IL-1β (10 ng/ml; Fujifilm Wako Pure Chemical Corp.), or IFN-β (500 ng/ml; Fujifilm Wako Pure Chemical Corp.).

### Transfection of small interfering (si)RNA

ESCs cells were transfected with either a non-targeting control, or with *SERPINA1*(EHU090971, Sigma-Aldrich), *TLR3* (EHU019541, Sigma-Aldrich), or *TLR4* siRNA (EHU086621, Sigma-Aldrich) using Lipofectamine RNAiMAX (Thermo Fisher Scientific) (22).

### RNA sequencing (RNA-seq), gene ontology (GO), and pathway analyses

RNA-seq analysis was performed with RNA extracted from cultured ESC lines using Isogen II (Nippon Gene). High-throughput sequencing libraries were prepared using a TruSeq Stranded mRNA LT Sample Prep Kit (Illumina, San Diego, CA, USA), according to the manufacturer’s instructions, and data analysis was performed by Macrogen Japan (Kyoto, Japan). Primary sequence data were deposited in the DDBJ (DNA Data Bank of Japan) Sequence Read Archive (https://www.ddbj.nig.ac.jp/dra/index-e.html; accession numbers: DRR304262 to DRR304264 and DRR304268 to DRR304270). Data analysis was performed as described previously (8). Briefly, trimmed sequences were analyzed using the TopHat/Cufflinks pipeline, the human genome (hg38), and reference annotations obtained from the UCSC genome browser (https://genome.ucsc.edu). Significantly differentially expressed genes (DEGs) were identified based on gene-level FPKM (fragments per kilobase of exon per million mapped fragments) expression levels. Genes that had an absolute expression level of >2 FPKM were selected. GO and Enriched Signaling Pathway analyses were performed using the Enrichr tool (http://amp.pharm.mssm.edu/Enrichr/).

### RNA extraction and quantitative RT-PCR

RNA was extracted from cultured cells using Isogen II (Nippon Gene), according to the manufacturer’s instructions, and reverse transcribed using a ReverTra Ace qPCR RT Kit (Toyobo, Osaka, Japan). The cDNA produced was then subjected to qPCR amplification using PowerUP SYBR Green Master Mix (Thermo Fisher Scientific). The primers are listed in **Table 1**. The amplification efficiency of each target gene and the reference gene, glyceraldehyde-3-phosphate dehydrogenase (GAPDH), was measured by calibration curves and found to be comparable. Sequence Detection System software v2.3 (Thermo Fisher Scientific) was used to determine the mean crossing threshold (Ct) values for each target (8).

**Table 1:** Primers for real-time PCR.

Name Accession No.		Sequence		Product length (bp)
GAPDH	F: 5’-	AGCCACATCGCTCAGACA	-3’	66
NM_002046.7	R: 5’-	GCCCAATACGACCAAATCC	-3’	
TLR3	F: 5’-	GCGCTAAAAAGTGAAGAACTGGAT	-3’	145
NM_003265.3	R: 5’-	GCTGGACATTGTTCAGAAAGAGG	-3’	
TLR4	F: 5’-	CCCTGAGGCATTTAGGCAGCTA	-3’	126
NM_138554.5	R: 5’-	AGGTAGAGAGGTGGCTTAGGCT	-3’	
MYD88	F: 5’-	GAGGCTGAGAAGCCTTTACAGG	-3’	129
NM_001172567.2	R: 5’-	GCAGATGAAGGCATCGAAACGC	-3’	
IRAK1	F: 5’-	TCAGAACGGCTTCTACTGCCTG	-3’	128
NM_001569.4	R: 5’-	TACCCAGAAGGATGTCCAGTCG	-3’	
IRAK2	F: 5’-	TCGAGTACCTGCATGGTCTGGA	-3’	114
NM_001570.4	R: 5’-	CAGGACACAGATGAGCCATTGG	-3’	
IRAK4	F: 5’-	ATGCCACCTGACTCCTCAAGTC	-3’	131
NM_001114182.3	R: 5’-	CCACCAACAGAAATGGGTCGTTC	-3’	
IRF1	F: 5’-	GAGGAGGTGAAAGACCAGAGCA	-3’	121
NM_002198.3	R: 5’-	TAGCATCTCGGCTGGACTTCGA	-3’	
IRF2	F: 5’-	TAGAGGTGACCACTGAGAGCGA	-3’	125
NM_002199.4	R: 5’-	CTCTTCATCGCTGGGCACACTA	-3’	
IRF3	F: 5’-	TCTGCCCTCAACCGCAAAGAAG	-3’	151
NM_001571.6	R: 5’-	TACTGCCTCCACCATTGGTGTC	-3’	
IRF7	F: 5’-	CCACGCTATACCATCTACCTGG	-3’	153
NM_001572.5	R: 5’-	GCTGCTATCCAGGGAAGACACA	-3’	
IRF9	F: 5’-	CCACCGAAGTTCCAGGTAACAC	-3’	123
NM_001385400.1	R: 5’-	AGTCTGCTCCAGCAAGTATCGG	-3’	
IL1B	F: 5’-	TGATGGCTTATTACAGTGGCAATG	-3’	140
NM_000576.3	R: 5’-	GTAGTGGTGGTCGGAGATTCG	-3’	
IL6	F: 5’-	CAGGAGCCCAGCTATGAACT	-3’	85
NM_000600.5	R: 5’-	AGCAGGCAACACCAGGAG	-3’	
CXCL8	F: 5’-	AAGCATACTCCAAACCTTTCCA	-3’	123
(NM_000584.4)	R: 5’-	CCAGACAGAGCTCTCTTCCA	-3’	
CXCL10	F: 5’-	GGTGAGAAGAGATGTCTGAATCC	-3’	134
NM_001565.4	R: 5’-	GTCCATCCTTGGAAGCACTGCA	-3’	
CXCL11	F: 5’-	AAGGACAACGATGCCTAAATCCC	-3’	116
NM_005409.5	R: 5’-	CAGATGCCCTTTTCCAGGACTTC	-3’	
IL12A	F: 5’-	TGCCTTCACCACTCCCAAAACC	-3’	100
NM_000882.4	R: 5’-	CAATCTCTTCAGAAGTGCAAGGG	-3’	
IFNB1	F: 5’-	CTTGGATTCCTACAAAGAAGCAGC	-3’	146
NM_002176.4	R: 5’-	TCCTCCTTCTGGAACTGCTGCA	-3’	
STAT1	F: 5’-	CCATCCTTTGGTACAACATGC	-3’	71
NM_007315.4	R: 5’-	TGCACATGGTGGAGTCAGG	-3’	
STAT2	F: 5’-	CAGGTCACAGAGTTGCTACAGC	-3’	118
NM_005419.4	R: 5’-	CGGTGAACTTGCTGCCAGTCTT	-3’	
STAT3	F: 5’-	CTTTGAGACCGAGGTGTATCACC	-3’	133
NM_139276.3	R: 5’-	GGTCAGCATGTTGTACCACAGG	-3’	
STAT5A	F: 5’-	GTTCAGTGTTGGCAGCAATGAGC	-3’	108
NM_012448.4	R: 5’-	AGCACAGTAGCCGTGGCATTGT	-3’	
ISG15	F: 5’-	CTCTGAGCATCCTGGTGAGGAA	-3’	136
NM_005101.4	R: 5’-	AAGGTCAGCCAGAACAGGTCGT	-3’	
ISG20	F: 5’-	ACACGTCCACTGACAGGCTGTT	-3’	137
NM_002201.6	R: 5’-	ATCTTCCACCGAGCTGTGTCCA	-3’	
MX1	F: 5’-	GGCTGTTTACCAGACTCCGACA	-3’	143
NM_001144925.2	R: 5’-	CACAAAGCCTGGCAGCTCTCTA	-3’	
MX2	F: 5’-	AAAAGCAGCCCTGTGAGGCATG	-3’	163
NM_002463.2	R: 5’-	GTGATCTCCAGGCTGATGAGCT	-3’	
APOBEC3G	F: 5’-	ATGACACCTGGGTCCTGCTGAA	-3’	114
NM_021822.4	R: 5’-	GAATCACGTCCAGGAAGCACAG	-3’	

F, Forward, R, Reverse.

### Western blot analysis

Cultured cells were lysed using RIPA buffer (Thermo Fisher Scientific). The constituent proteins were separated by SDS-PAGE and transferred onto polyvinylidene difluoride membranes (Bio-Rad Laboratories, Hercules, CA, USA) using a trans-Blot Turbo (Bio-Rad). After blocking with Bullet Blocking One (Nacalai Tesque, Inc., Kyoto, Japan), the membranes were incubated with primary antibodies specific for SERPINA1 (1:2,000; Dako, Tokyo, Japan), TLR3 or TLR4 (1:2,000; Proteintech, Tokyo, Japan), MYD88, IRAK1, IRAK4, phosphorylated (p)-IRAK4, IκB, p-IκB, NF-κB, p-NF-κB, IL-1β, COX2, STAT3, JNK, p-JNK, p38, p-p38, ERK1/2, p-ERK1/2 (1:2,000; Cell Signaling Technology, Beverly, MA, USA), MX1 (1:2,000; Sigma-Aldrich), ISG15 (1:2,000; Origene Technologies, Rockville MD, USA), and GAPDH (1:5,000, Fujifilm Wako Pure Chemical Corp.). Immunoreactive bands were detected using an enhanced chemiluminescence kit (Merck Millipore, Burlingame, MA, USA) after incubation with horseradish peroxidase-labeled goat anti-rabbit or anti-mouse IgG (1:5,000; Vector Laboratories, Burlingame, CA, USA). Signals were detected using a C-DiGit Blot Scanner (LI-COR, Lincoln, NE, USA) (22).

### Enzyme-linked immunoassay (ELISA)

Culture media were centrifuged at 10,000 × g at 4°C for 10 min and the supernatants were concentrated using a Micron filter device (Amicon ultra-0.5 centrifugal filter unit; Merck Millipore). The concentrations of IL-1β, IL-6, and IL-8 (encoded by the *CXCL8* gene) in the medium were measured using an ELISA kit (Human IL-6 Simple Step ELISA Kit; Abcam, Tokyo, Japan). The concentration of IFN-β in the supernatant was also measured using an ELISA kit (Human IFN-beta Sandwich ELISA kit; Proteintech) (7).

### Immunohistochemistry

Endometriotic tissue was obtained from three patients who were undergoing surgery. The protocol was approved by the Clinical Research Ethics Committee of Saitama Medical University and Tokyo University of Pharmacy and Life Sciences (#1512). Paraffin sections of endometriotic tissue were immunostained with antibodies targeting TLR3, TLR4, MYD88, E-cadherin, or cytokeratin, according to a previously described protocol (8). Briefly, paraffin sections were rehydrated, boiled for 20 min in 10 mM citrate buffer (pH 6.0), and then incubated overnight at 4°C with antibodies specific for TLR3 (1:200; Proteintech), TLR4 (1:200; Proteintech), MYD88 (1:200; Cell Signaling Technology), E-cadherin (1:200; Cell Signaling Technology), or cytokeratin (1:100; Dako). Subsequently, sections were incubated with Alexa Fluor 568- or 488- labeled alpaca anti-rabbit IgG or anti-mouse IgG2b (Life Technologies Corporation, Carlsbad, CA, USA). Nuclear counterstaining was performed using 4’,6-diamidino-2-phenylindole (DAPI; Life Technologies Corporation). The fluorescently labeled cells were examined under a BZ-X800 microscope (Keyence, Osaka, Japan).

### Statistical analysis

The qPCR data represent the results of three or more independent experiments, with each sample assayed in triplicate. Data are expressed as the mean ± SEM and were compared using Dunnett’s test in R software (v4.0.5). *P* < 0.05 was considered statistically significant. In RNA-seq analysis, a false discovery rate-adjusted *P*-value (*q*-value) <0.05 was considered statistically significant (8).

## Results

### Knockdown of *SERPINA1* upregulates expression of TLRs by ESCs

**Figure 1:**
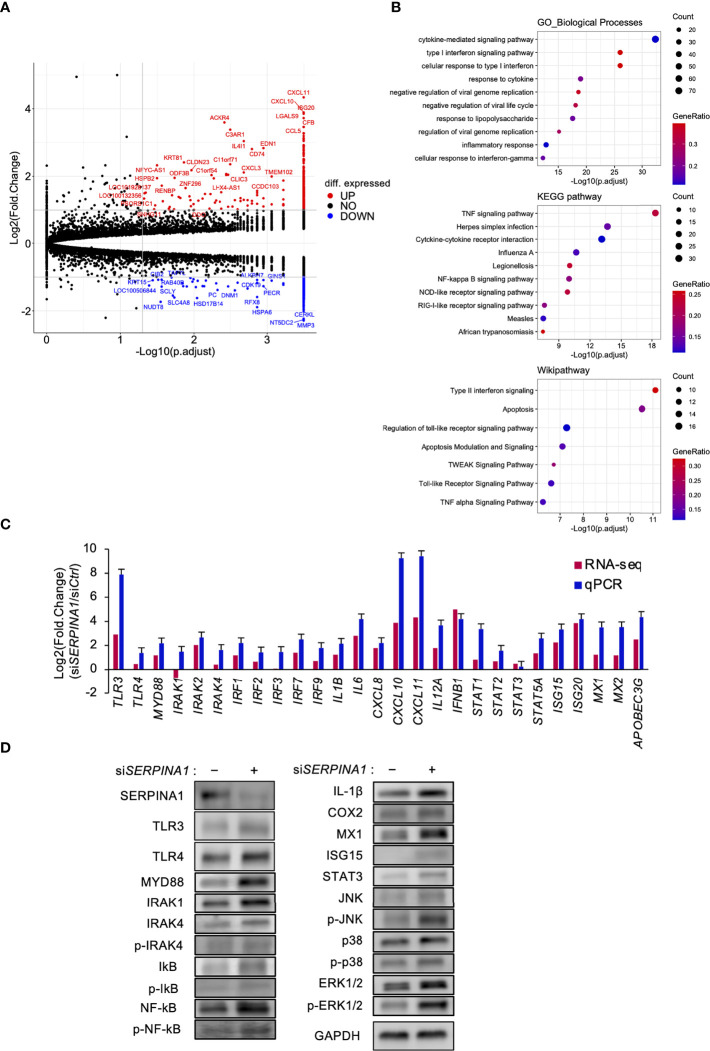
Knockdown of *SERPINA1* upregulates expression of TLRs in ESCs. ESC lines were transfected for 24 h with siRNA specific for *SERPINA1*. Next, RNA was extracted and subjected to RNA sequencing (RNA-Seq; **A, B**). **(A)** Volcano plot showing expression of transcripts identified by RNA-seq. The transcripts highlighted in red or blue were differentially expressed by 2-fold (*P* < 0.05). **(B)** Differentially expressed genes were functionally classified using Gene Ontology analysis with the biological process, KEGG pathway, or Wikipathway datasets. **(C)** Expression of Toll-like receptor-related (*TLR3/4, IRAKs*, and *IRFs*), inflammatory-related (*ILs* and *CXCLs*), or interferon-related (*IFNB1, STATs, ISGs, MXs*, and *APOBEC3G*) genes in primary ESCs was measured by qPCR (n=3). *GAPDH* mRNA was used as the reference gene. Values represent the mean ± SEM of three independent experiments. **(D)** Lysates prepared from primary ESCs transfected for 24 h with si*SERPINA1* were subjected to immunoblotting. GAPDH served as a loading control.

To explore the effect of knocking down *SERPINA1* on gene expression in ESCs, we performed RNA-seq analysis. This identified 760 DEGs, of which 337 were downregulated and 423 were upregulated (**Figure 1A**). KEGG pathway, Wikipathway, and GO enrichment analyses of upregulated genes identified those related to inflammatory-related signals, including “TNF signaling pathway”, “Interferon signaling”, and “Toll-like receptor signaling pathway” (**Figure 1B**). The qPCR also revealed an effect of *SERPIN1* knockdown on expression of inflammatory-related genes, TLR signaling factors, and interferon stimulated genes (ISGs) in ESCs, which were similar to those measured by RNA-seq analysis (**Figure 1C**). Furthermore, expression of TLR3, TLR4, MYD88, IRAK1, IL-1β, COX2, MX1, STAT3, IκB, and NF-κB proteins, and phosphorylation of IRAK4, MAPKs (JNK, p38, and ERK1/2) were appeared to be higher in ESCs knocked down for *SERPINA1* (**Figure 1D**), supporting to the results of RNA-seq and qPCR analysis.

### TLR3 upregulates expression of TLR- or inflammatory-related genes in ESCs

**Figure 2:**
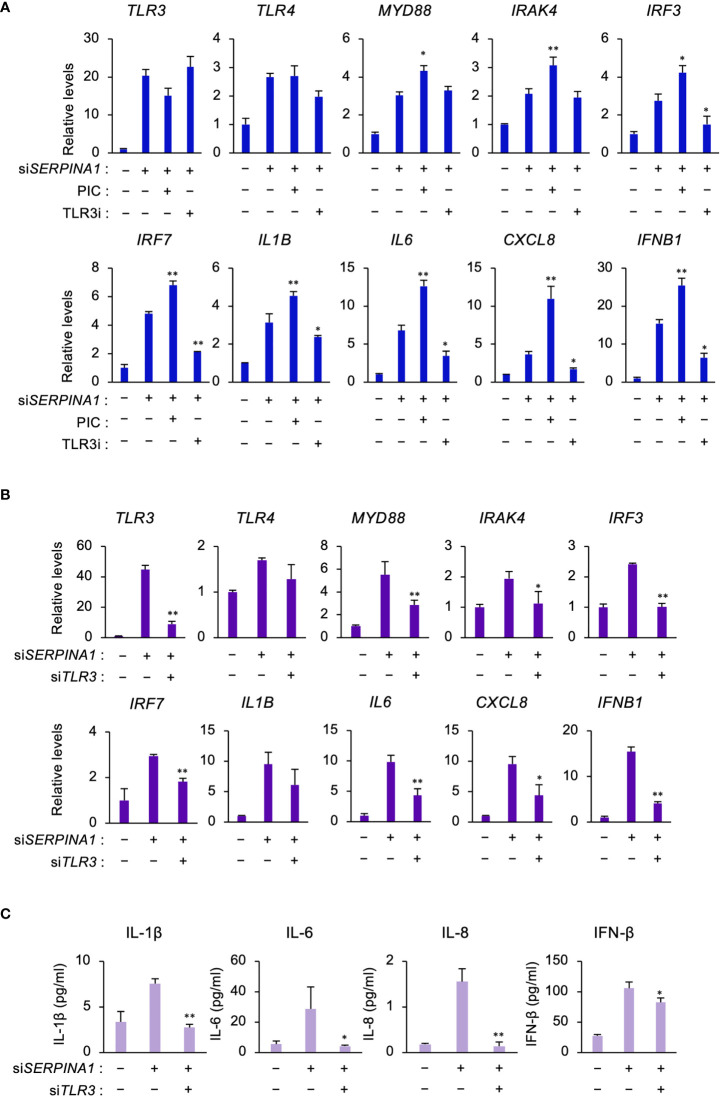
TLR3 upregulates expression of TLR- or inflammatory-related genes in ESCs. **(A)** Expression of *TLR3*, *TLR4, MYD88, IRAK4, IRF3, IRF7, IL1B, IL6*, *CXCL8, and IFNB1* in primary ESCs pre-treated with PIC (10 μg/ml) or TLR3i (10 μM) and then transfected for 24 h with si*SERPINA1* (n=3). *GAPDH* was used as the reference gene. Values represent the mean ± SEM of three independent experiments. *P<0.05, **P<0.01 vs. si*SERPINA* alone. **(B, C)** Primary ESCs transfected for 24 h with si*SERPINA1* and/or si*TLR3*. **(B)** Expression of *TLR3*, *TLR4, MYD88, IRAK4, IRF3, IRF7, IL1B, IL6*, *CXCL8, and IFNB1* in primary ESCs (n=3). *GAPDH* was used as the reference gene. Values represent the mean ± SEM of three independent experiments. *P<0.05, **P<0.01 vs. si*SERPINA* alone. **(C)** The culture media were subjected to the ELISA to measure the concentration of IL-1β, IL-6, IL-8, or IFN-β secreted from primary ESCs. Values represent the mean ± SEM of three independent experiments. *P<0.05, **P<0.01 vs. si*SERPINA1* alone.

To determine whether TLR3 signaling regulated the expression of TLR signaling- and inflammatory-related DEGs in *SERPINA1*-knockdown ESCs, cells were treated with a TLR3-selective stimulator (PIC) or inhibitor (TLR3i). PIC further increased si*SERPINA1*-induced expression of *MYD88, IRAK4, IRF3, IRRF7, IL1B, IL6, CXCL8*, and *IFNB1* (**Figure 2A**). By contrast, TLR3i decreased the expression of all these genes, except for *MYD88* and *IRAK4* (**Figure 2A**). Furthermore, knockdown of TLR3 using specific *TRL3* siRNA decreased si*SERPINA1*-induced TRL signaling and the expression of inflammatory-related genes in ESCs (**Figure 2B**). Similar to mRNA expression, si*SERPINA1*-induced secretion of IL-1β, IL-6, IL-8, and IFN-β by ESCs decreased after TLR3 knockdown (**Figure 2C**).

### TLR4 increases expression of inflammatory-related genes in SERPINA1-silenced ESCs

**Figure 3:**
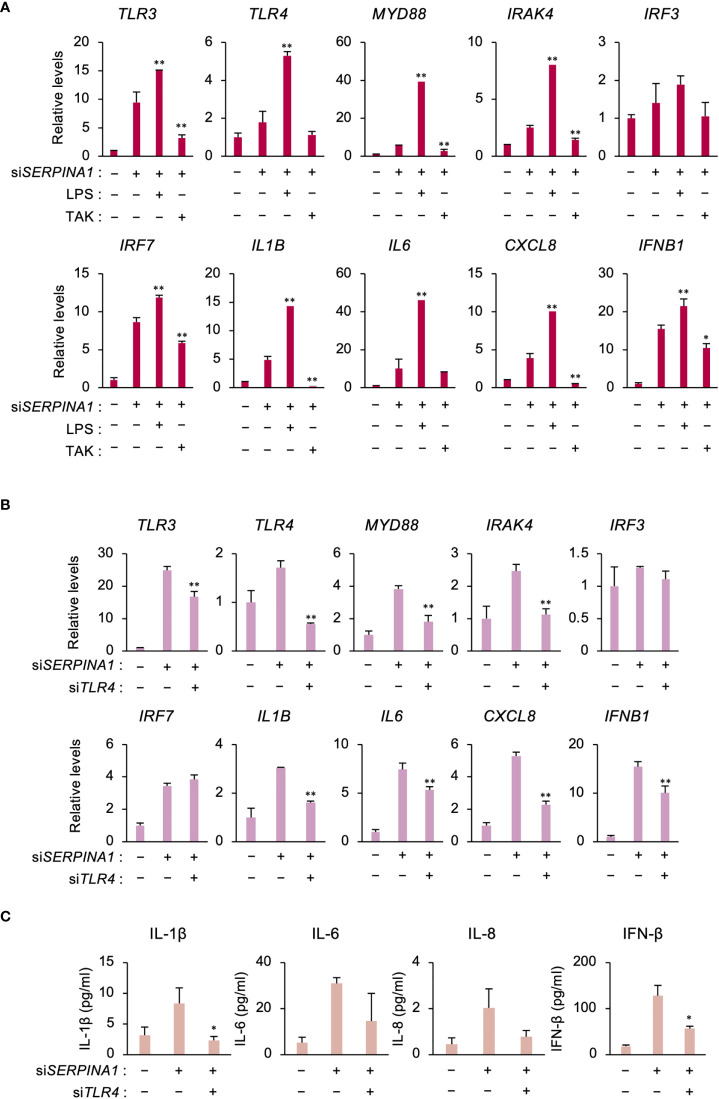
TLR4 increases expression of inflammatory-related genes in SERPINA1-silenced ESCs. **(A)** Expression of *TLR3*, *TLR4, MYD88, IRAK4, IRF3, IRF7, IL1B, IL6*, *CXCL8, and IFNB1* in primary ESCs pre-treated with LPS (0.2 μg/ml) or TAK (10 μM) and then transfected for 24 h with si*SERPINA1* (n=3). *GAPDH* was used as the reference gene. Values represent the mean ± SEM of three independent experiments. *P<0.05, **P<0.01 vs. si*SERPINA1* alone. **(B, C)** Primary ESCs transfected for 24 h with si*SERPINA1* and/or si*TLR4*. **(B)** Expression of *TLR3*, *TLR4, MYD88, IRAK4, IRF3, IRF7, IL1B, IL6*, *CXCL8, and IFNB1* by ESCs (n=3). *GAPDH* was used as the reference gene. Values represent the mean ± SEM of three independent experiments. *P<0.05, **P<0.01 vs. si*SERPINA1* alone. **(C)** Culture media were subjected to the ELISA to measure the concentration of IL-1β, IL-6, IL-8, or IFN-β secreted from primary ESCs. Values represent the mean ± SEM of three independent experiments. *P<0.05 vs. si*SERPINA1* alone.

Similar to TLR3, RNA-seq analysis identified TLR4 as an upregulated DEG in *SERPINA1*-knockdown ESCs. Therefore, to examine the effect of TLR4 on the expression of TLR signaling- and inflammatory-related factors, cells were treated with a TLR4-selective stimulator (LPS) or inhibitor (TAK). LPS further increased si*SERPINA1*-induced expression of TLR signaling- and inflammatory-related genes, except for *IRF3* (**Figure 3A**). Conversely, TAK decreased expression of these genes, with the exception of *IRF3* and *IL6* (**Figure 3A**). Furthermore, TLR4 knockdown by siRNA decreased si*SERPINA1*-induced expression of TRL signaling- and inflammatory-related genes (except *IRF3* and *IRF7)* in ESCs (**Figure 3B**). Similar to mRNA expression, si*SERPINA1*-induced secretion of IL-1β and IFN-β by ESCs was decreased by TLR4 knockdown, while the secretion of IL-6 and IL-8 also tended to decrease (**Figure 3C**).

### IL-1β or IFN-β increases expression of inflammatory-related genes

**Figure 4:**
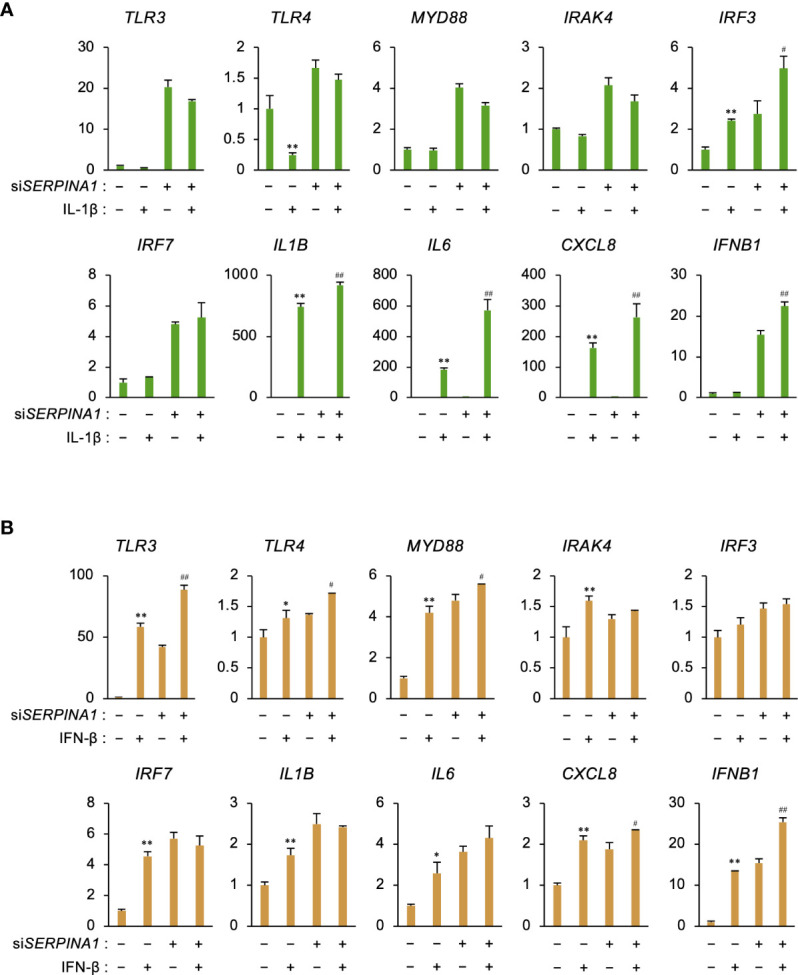
IL-1β or IFN-β increases expression of inflammatory-related genes. **(A, B)** Expression of *TLR3*, *TLR4, MYD88, IRAK4, IRF3, IRF7, IL1B, IL6*, *CXCL8, and IFNB1* in primary ESCs transfected for 24 h with si*SERPINA1* and then treated with IL-1β (**A**; 10 ng/ml) or IFN-β (**B**; 500 ng/ml) (n=3). *GAPDH* was used as the reference gene. Values represent the mean ± SEM of three independent experiments. *P<0.05, **P<0.01 vs. Ctrl. ^#^ *P*<0.05, ^##^ *P*<0.01 vs. si*SERPINA1* alone.

To examine whether secreted IL-1β or IFN-β affects ESCs in an autocrine fashion, cells were treated with recombinant IL-1β or IFN-β. IL-1β further increased si*SERPINA1*-induced expression of *IRF3, IL6, CXCL8*, and *IFNB1* (**Figure 4A**). In addition, IFN-β increased si*SERPINA1*-induced expression of *TLR3, TLR4, MYD88, CXCL8*, and *IFNB1* (**Figure 4B**).

### TLR3, TLR4, and MYD88 localize at ESCs in endometriosis lesions

**Figure 5:**
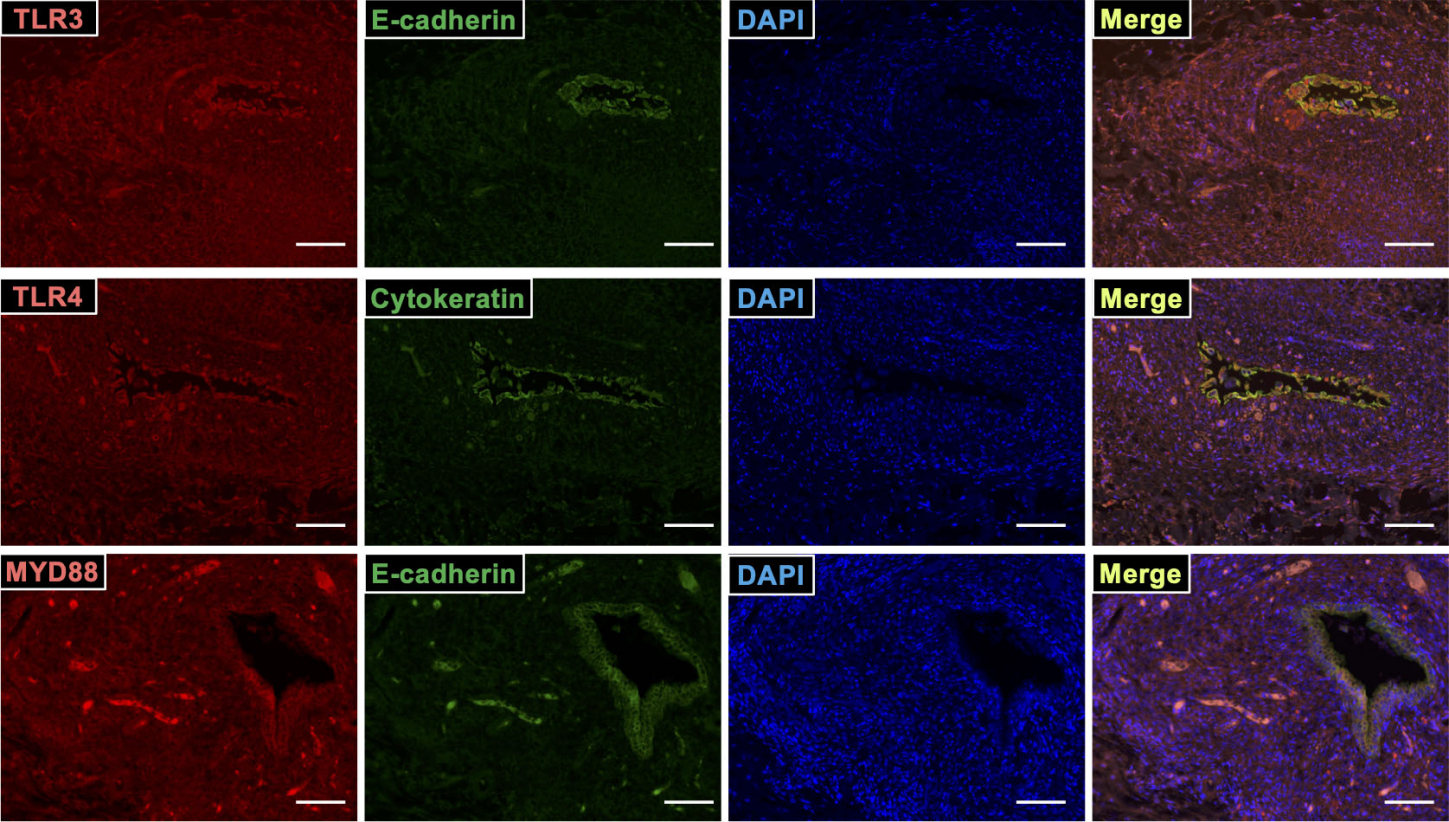
TLR3, TLR4, and MYD88 localized at ESCs in endometriosis lesion. Sections of endometriotic tissue were immunostained for TLR3, TLR4, MYD88 (red), and epithelial markers (E-cadherin and cytokeratin; green), and then counterstained with DAPI. Scale bar = 100 μm.

Localization of TLR3, TLR4, and their downstream factor MYD88, was examined in endometriotic lesions using immunohistochemistry. TLR3, TLR4, and MYD88 proteins localized to stromal cells, and to E-cadherin- or cytokeratin-stained glandular epithelial cells (**Figure 5**).

## Discussion

**Figure 6:**
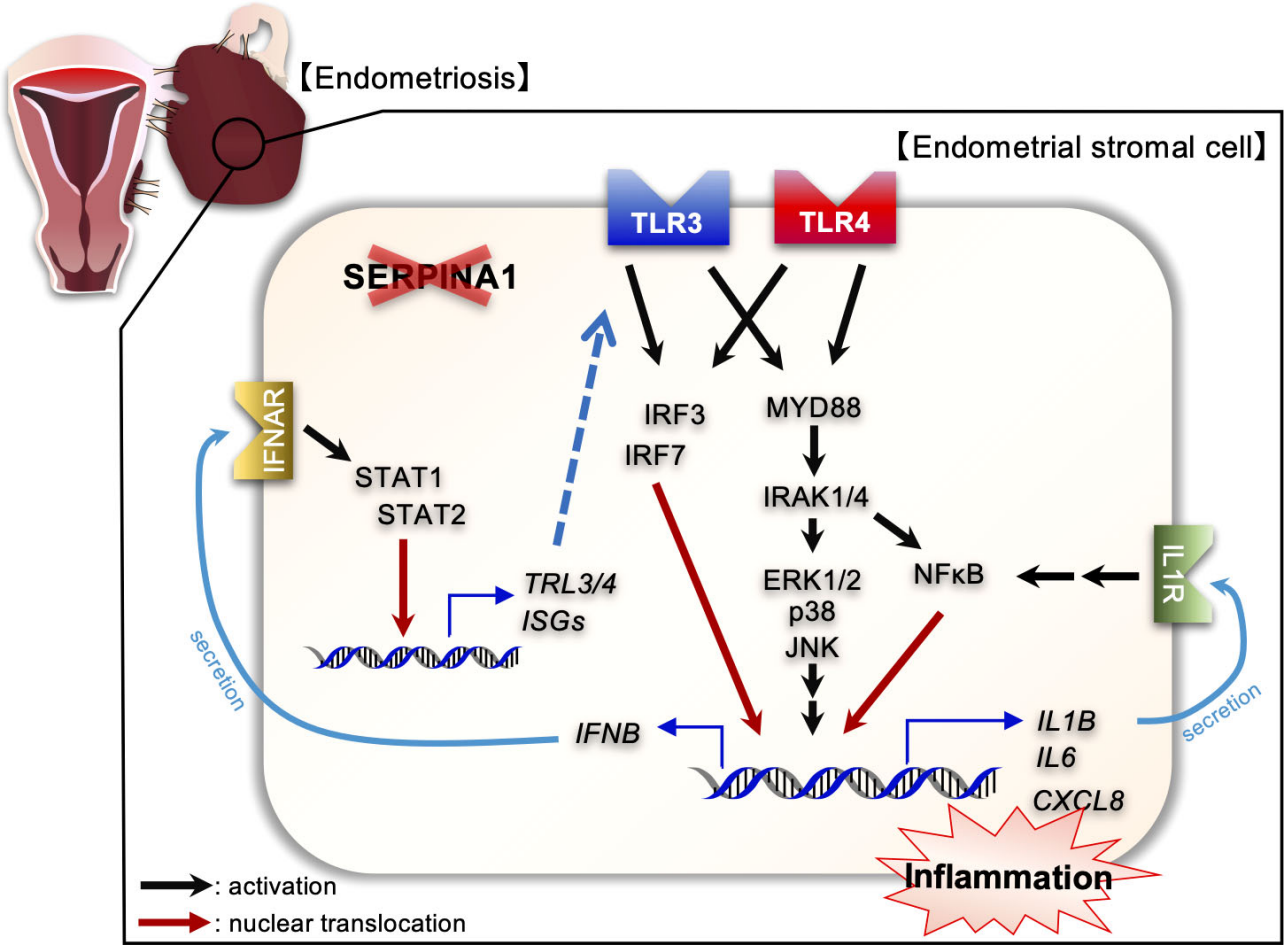
Diagram illustrating the effects of SERPINA1 knockdown on expression of inflammatory-related factors *via* TLR3/4 associated with endometriotic human endometrial stromal cells. SERPINA1 knockdown induces and activates TLR3 and TLR4 signaling, leading to production of inflammatory cytokines, of which IL-1β or IFN-β promote expression of inflammatory-related genes in an autocrine fashion.

The present study is the first to report global transcriptome analysis of SERPINA1-knockdown ESCs. Enrichment analyses of upregulated DEGs revealed that knocking down SERPINA1 affected inflammatory cytokine signaling pathways, including TLRs, IFN, and tumor necrosis factor (TNF). Furthermore, we also noted an increase in the amounts of TLR3 and TLR4 proteins, along with increased expression and phosphorylation of their downstream factors in SERPINA1 knocked-down ESCs. Therefore, we used stimulators, inhibitors, or siRNA to examine the effects of TLR3/4 on expression of inflammatory cytokines and TLR signaling factors MYD88/IRAK4 and IRF3/7 by SERPINA1-silenced ESCs. Treatment with PIC increased expression of these factors, whereas a TLR3 inhibitor decreased expression. In addition, si*TLR3* reduced secretion of IL-1β, IL-6, IL-8, and IFN-β by ESCs, possibly through downregulation of MYD88/IRAK4 and IRF3/7. Similar to TLR3, the TLR4 agonist LPS increased si*SERPINA1*-induced expression of TLR signaling- and inflammatory-related genes, whereas a TLR4 inhibitor (TAK) reduced their expression. Furthermore, transfection of si*TLR4* reduced secretion of IL-1β and IFN-β. These results indicate that inhibiting expression of si*SERPINA1* induces production of inflammatory cytokines *via* an increase in TLR3/4 proteins, and subsequent activation of their downstream effectors MYD88/IRAK/NF-κB and IRF3/7 (**Figure 6**).

TLRs are transmembrane proteins comprising three domains: an extracellular domain (responsible for recognition of pathogen-associated molecular patterns (PAMPs)) and danger-associated molecular patterns (DAMPs); a transmembrane domain; and an intracellular domain required for downstream signaling transduction (24, 25). Activation of TLRs triggers a signaling cascade that leads to transcription of numerous genes involved in inflammation and anti-microbial defense. To date, the mammalian family of TLRs comprises 13 members. TLRs 1–9 are conserved between humans and rodents (24, 25). Differences between TLRs include ligand specificity, expression patterns, and activation of different signaling pathways and cellular responses. The endometrium expresses TLRs involved in production of proinflammatory cytokines such as IL-1β. Several studies reported that TLRs may play a role in endometriosis (26). Expression of TLR3/4 in the endometrium of women with endometriosis is higher than that in healthy women (27–33). Furthermore, aberrant TLR activation by DAMPs, which may be induced by oxidative stress and inflammation, during the retrograde menstruation might lead to chronic inflammation and anti-apoptotic responses in endometriotic lesions (26, 34). Our study also found upregulation of TLR3 and TLR4 in SERPINA1-silenced ESCs. Because expression of SERPINA1 is decreased in endometriosis-like lesions in a mouse model, it is possible that TLRs may be involved in inflammatory responses in these lesions. Of note, SERPINA1 knockdown activated TLR3 and TLR4 independently of the treatment with their respective stimulators, as demonstrated by the experiments with TLR3 or TLR4 inhibitors and specific siRNAs. These findings suggest that ESCs in which endometrial SERPINA1 is silenced could induce development and/or exacerbation of endometriosis, independently of microbial/viral infection. However, the molecular mechanisms by which a decrease in SERPINA1 expression upregulates and activates TLR3/4 in ESCs remains unclear; therefore, further investigations are needed to identify the role of SERPINA1 in ESCs.

In the present study, we showed that SERPINA1 knockdown induces inflammatory cytokines such as IL-1β, IL-6, IL-8, and IFN-β through TLR3/4 activation, followed by activation of the MYD88/IRAK1/4 and IRF3/7 pathways. TLR3/4 regulates several pathways, including MYD88 and TRIF, followed by IRF3/7, MAPKs, and NF-κB, leading to production of cytokines such as IL-1β and IFN-β (26, 35). Recent studies have shown that inflammasomes are involved in intracellular pathways associated with inflammation (36–38). Inflammasomes respond to several PAMPs, including LPS and DAMPs such as uric acid crystals. Nucleotide-binding oligomerization domain-like receptors (NLRs) are intracellular sensors of PAMPs and DAMPs. A representative NLR, NLRP3, is characterized by the presence of a pyrin domain. Activation of NLRP3 promotes its oligomerization, which leads to recruitment and clustering of the inflammasome adapter apoptosis-associated speck-like protein and caspase-1 protease, leading to caspase activation. Activated caspase-1 promotes maturation of IL-1β. COX2 may play an important role in PGE2-mediated activation of the NLRP3 inflammasome (39). Indeed, the NLRP3 inflammasome induces caspase-1 and IL-1β in endometriosis (40, 41). A previous study reported that knockdown of SERPINA1 induces expression of NLRP3, several inflammatory cytokines (IL-1β, IL-6, IL-8), and COX2 in human adipocytes (16). The present study showed that knockdown of SERPINA1 induces IL-1β, and that treatment of ESCs with IL-1β further increases expression of inflammatory cytokines. These findings may imply that silencing stromal SERPINA1 generates IL-1β production in endometriotic lesions *via* mRNA expression induced by TLRs/MYD88/NF-κB, along with NLRP3 inflammasome-induced caspase-1, all of which may cause a chronic inflammatory response in an autocrine or paracrine fashion.

Our RNA-seq analysis of SERPINA1-knockdown ESCs also identified interferon signaling pathways in addition to inflammatory-related signals. SERPINA1 knockdown increased production of IFN-β *via* TLR3/4 activation. Notably, TLR3/4 increases production of IFN-β through MYD88 and TRIF signaling (35). Like IL-1β, we examined the possibility that secreted IFN-β affects ESCs in an autocrine or paracrine fashion. Interestingly, we found that IFN-β upregulated TLR3/4 expression in addition to expression of inflammatory cytokines and ISGs. These data suggest that enhanced IFN-β production may upregulate TLR3/4 expression, thereby enhancing TLR3/4 signaling and exacerbating the inflammatory characteristics of endometriosis.

Taken together, the results presented herein suggest that inhibiting expression of SERPINA1 in ectopic ESCs induces expression of inflammatory factors associated with TLR3/4, IL-1β, and IFN-β signaling (**Figure 6**). Further studies are needed to identify the molecular mechanisms underlying the interaction between SERPINA1 and the TLR3/4, IL-1β, and IFN-β signaling pathways. Such studies might lead to identification of novel treatments for endometriosis.

## Data availability statement

The original contributions presented in the study are publicly available. This data can be found here: DNA Data Bank of Japan (DDBJ), Sequence Read Archive (https://www.ddbj.nig.ac.jp/dra/index-e.html). Accession numbers are as followed: DRR304262, DRR304263, DRR304264, DRR304268, DRR304269, DRR304270.

## Ethics statement

The studies involving human participants were reviewed and approved by Clinical Research Ethics Committee of Tokyo Medical University Hospital Clinical Research Ethics Committee of Saitama Medical University and Tokyo University of Pharmacy and Life Sciences. The patients/participants provided their written informed consent to participate in this study.

## Author contributions

KK and AS contributed to data acquisition. KK and AS contributed to data analysis. MA, MY, YM, and TK prepared tissue sections. MY, JK, MO, and HN isolated primary endometrial stromal cells. KK and KT contributed to study conception, design, and manuscript preparation. All authors contributed to the article and approved the submitted version.

## Funding

This research was funded by KAKENHI Grants-in-Aid for Scientific Research [numbers 20H03133 to KK, and 22K09651 to KT] from the Japan Society for the Promotion of Science.

## Conflict of interest

The authors declare that the research was conducted in the absence of any commercial or financial relationships that could be construed as a potential conflict of interest.

## Publisher’s note

All claims expressed in this article are solely those of the authors and do not necessarily represent those of their affiliated organizations, or those of the publisher, the editors and the reviewers. Any product that may be evaluated in this article, or claim that may be made by its manufacturer, is not guaranteed or endorsed by the publisher.
